# Critical Analysis of Select Hospital Reports in the Lancet

**Published:** 1827-10

**Authors:** 


### CRITICAL ANALYSIS

OF

### SELECT HOSPITAL REPORTS

IN THE

### LANCET.

No. 194, May 19th. } *Acupuncture in Sciatica.*

Scotus reports a case from the Edinburgh Royal Infirmary, in which, sciatica had resisted all the usual modes of treatment, and where the needle was three times introduced, with real or supposed relief for the time; but, as the pain always returned, acupuncture was abandoned. Dr. Graham even became witty on this occasion, and compared the "seat of honour," in the poor patient's case, to a pincushion! It seems that the *wise men of the North* consider acupuncture as one of those French bubbles, (like the stethoscope, for example) which the effervescence of invention has cast on the stream of public opinion, to float for a short time, and then disappear. But it should be recollected that this is not a French invention, but one derived from their brethren, the *wise men of the East*, who, in their maps of the world, represent their own country as a large continent, and all the other countries of the world, for example, France, Germany, Spain, America, England, &c. as little islands, studded, at various distances, in the ocean around the celestial empire! We fear that this *celestial* empire is not confined to China or Japan. But—VERBUM SAT.

*Hernia.* Two cases of this disease are reported from Bartholomew's.

*Case* 1. This patient had been subject to hernia for 17 years, but manipulation had always succeeded in the reduction till this time, when it failed. The man was instantly sent to the hospital, and was seen in an hour after the strangulation; yet the tumour in the groin was so exceedingly tender and painful, that the taxis could not be borne. "A warm bath was ordered, and the *arm* speedily placed in it. Twenty-eight ounces of blood were then taken from *the arm*, but syncope was not induced. The tobacco-injection was then given, and vomiting succeeded. "The matter thrown up possessed not the slightest *fæcal odour*, on which Mr. Charles Bell places great reliance, as denoting the *certain existence* of incarceration." The injections and attempts at taxis having failed, the operation was performed. The hernia was congenital, consisting of omentum, and six or seven inches of *small intestine*. These were returned, and the patient did well.

We have often been surprized that surgeons should consider *stercoraceous* vomiting to be a necessary consequence, or certain proof, of strangulation of the gut. How can stercoraceous matters return from the large intestines, (where alone they can acquire the "*fæcal odour*") if a portion of small intestine, as in the above case is incarcerated? The thing is absurd.

The second case, which proved fatal, seems to be chiefly introduced for the purpose of throwing odium, without any proof, on some member of the Royal College of Surgeons, for imputed neglect in the treatment of a scrotal hernia. The practitioner was called up after three in the morning, and attempted the taxis, but failed, and then bled the patient. Again the reduction was attempted. At eight o'clock in the morning, the man was sent to Bartholomew's, and his death is evidently imputed to the few hours delay which intervened. We are no advocates for procrastination in cases of strangulated hernia; but there are few practitioners, we believe, who would venture to operate in five hours from the time a hernia came down:—And we do hope that there are few, except the reporters in the Lancet, who would endeavour to affix a stain on the professional character of one of their brethren for such a delay. When will this diabolical system of literary assassination cease to be relished by a profession styling itself learned and liberal?

Mr. Vincent operated, but the patient sunk, in two days, under general peritoneal inflammation, and gangrene of the portion of ileum that had been incarcerated. This case offers an illustration of the danger of delay in some cases of hernia; but the difficulty of discriminating these cases from those in which the taxis and other means may be much longer employed, is great indeed.

---

No. 195, May 26th. } *Softening of the Brain.*

Under the head of "HOSPITAL SURGERY," Panton Square, there is a note of a dissection in Mr. Wardrop's practice, exemplifying that pathological condition, now so well known, especially on the Continent, by the term *ramollissement.*

Three weeks before the patient's death, he had been exposed to great mental excitement, became very irascible, and uncontrollable in these paroxysms. He consulted Mr. Wardrop for pain in his forehead, which was constant, though variable; pulse unaffected, except in frequency; tongue loaded; skin warm; bowels irregular. He was purged and cupped, at first with temporary relief; but the pain returned with augmented force, the pulse being feeble and compressible. In a few days, he complained of loss of power in the left arm and leg, followed by difficulty of utterance, and feebleness of the right side. General and local bleeding produced no relief, and he soon lost the power of both sides of the body. His intellect was unimpaired, and he died in a few days after the supervention of the general paralysis.

*Dissection.* The dura mater had lost its white and glistening appearance, and was thickened, in some places, with marks of former inflammation. There was some water in the ventricles—pia mater very vascular—tuberculum annulare, in some places, quite soft and pulpy, yielding to the slightest pressure under the finger. In fact, the greater portion of it was in a state of deliquescence.

---

No. 198, June 16th. } *Congenital Division of the Palate.*

We regret to see that the operation for this defect was unsuccessfully performed by Mr. Wardrop and Mr. Alcock. The patient was 21 years of age, and her soft palate and uvula were divided into two symmetrical portions, the bones being entire. Three broad ligatures were first introduced through the soft palate at regular distances—the callous edges cut off—and the edges brought (but not to the very end) into contact. There was much hæmorrhage and no adhesion.

*Abscess of the Liver.*—We are greatly inclined to think there is a misnomer here. A man, aged 57, presented himself in Panton Square, "with a globular tumour, the size of the fœtal head, extending in the course of the linea alba, from the ensiform cartilage nearly to the umbilicus. The swelling was soft and *elastic*, and afforded a distinct, though deeply-seated sense of fluctuation." There was little pain on pressure, and the colour of the integuments unaffected. There was constant uneasiness in the loins and right shoulder, with occasional rigors—skin yellow, stools white. This swelling was first noticed after an attack of jaundice, with acute symptoms, and had rapidly increased. Mr. Wardrop had no doubt it was an abscess in the liver, or connected with that viscus. "In these affections, he observed, it is much better to avoid making any opening, with the view of discharging the purulent matter, for he was sure he had seen many bad consequences arise from puncturing *swellings* of a similar nature." Acting on this principle, some calomel and rhubarb were ordered every night. "A few days ago the man returned to shew himself: his general health is greatly improved. The tumour has now *nearly disappeared,*

and no sense of fluctuation can be perceived." He had passed nothing particular by stool, nor has he had any expectoration.

*Remarks.* In the first place, we question the fact of this being an hepatic abscess. We have seen many cases of this disease, but never saw, or heard, or read of any one, *the size of a fœtal head*, going off in such a manner, without sensible effects while bursting into the cavity of the abdomen, chest, stomach, or colon. It is more likely to have been an enlargement of the gall-bladder from obstruction of the ducts.* We lately saw an instance of this kind, where a patient died of disease of the liver, the gall-bladder being capable of holding a pint of fluid. Or (what is more likely) the case might have been an hydatid—but human experience gives it against abscess.

And, secondly; granting that it was an abscess, we hold it to be one of the best established principles in physic or surgery, to give external vent to an hepatic abscess, presenting a globular tumour the size of a child's head, with distinct fluctuation. Can it be expected that such a collection of pus can be absorbed? Certainly not. The other terminations then must, in all probability, be by bursting internally. In respect to the case in Panton Square, however, we perfectly agree with Mr. Wardrop that there might be much mischief done "from puncturing *swellings* of a similar nature."

*Curious Appearance in a Stump.*—A boy's arm had been amputated some years ago, by Dr. Thompson, and the stump, well covered, had soon healed. The boy went into the country, and was lost sight of for some years. He lately returned, with the stump presenting a very conical and tapering form. No muscular substance covers the bone within two inches and a half of its extremity. Three quarters of an inch of the bone projects completely beyond the skin. This projection has become covered with a horny substance exactly resembling the finger nail, and in the form of a thimble. The patient suffers no pain, but is obliged to keep the stump well covered to defend it from injury. Mr. W. intends to amputate this projecting portion.

No. 199, June 23rd. } *Carcinomatous Disease of the Palate.*

This case is (at the time of report) in St. Thomas's Hospital. The following is the state of the parts. A man, 56 years of age, had a tooth extracted some time previously, and was informed by the surgeon, that he had a disease of the mouth, for which he was recommended to Bartholomew's Hospital. He remained there a few weeks, and then left it. He entered St. Thomas's Hospital on the 3rd of May. On looking into the mouth, the right side of the palate was found covered with florid, hard granulations—the same appearance extending over the jaw, and affecting the cheek—extending backwards and involving a portion of the velum pendulum palati. The reporter avers that there is no distinct breach of surface, "but simply a conversion of the naturally smooth and polished condition of the mucous membrane into a surface covered with rugged substances, fleshy in appearance and resembling granulations, but of cartilaginous hardness." The parts are but little sensible, and the patient experiences little or no pain in the mouth. The glands beneath the jaw are enlarged, and of stony hardness, attended with occasional lancinating pains there. There were two other subcutaneous tumours on the abdomen, and one on the chest, the precise nature of which is not ascertained. Sarsaparilla, soda, and anodynes, are the medicines prescribed. The man's health is not bad. A solution of nitrate of silver is ordered to the parts.

No. 200, June 30th, } STRANGULATED HERNIA.

W. Bates, æt. 43, was brought into Bartholomew's Hospital, April 1st, at four p. m., labouring under nausea, hiccup, vomiting, abdomen tense, and rather painful on pressure. In the left groin, just over Poupart's ligament, was a small

* "Une femme mourut de consumption: à l'ouverture du cadavre, on trouva les deux conduits biliaires completement interceptés, le cystique par un calcul—l'hepatique un tubercule developpé dans le foie. *La vesicule du fiel était tellement distendue par la bile, qu'elle égalait l'estomac en volume.*"—MAGENDIE REVUE MÉDICALE, MAI, 1827, p. 260.

tumour, which was rather painful; pulse 120 small, and weak. He states, that between two and three months ago, the tumour first appeared, that since that time the bowels have been irregular, and that on the 28th of March, the bowels being costive, he was attacked with nausea and vomiting, whilst the tumour became larger and more painful. On the three succeeding days he took some castor oil, &c. which was immediately rejected, and just before his admission he was bled by a surgeon, who employed the taxis and succeeded in reducing the tumour to half its previous size. He has had no motion since the 27th.

He was now put into the warm bath, and reduction attempted, but without effect. *Calomel and jalap—enema.* The medicine was rejected, and the glyster brought away no fæcal matter. At 9 P. M. Mr. Lloyd saw the patient, but not being quite satisfied of the existence of hernia, he ordered V. S. ad ℥xvj. with five grains of calomel and a grain and a half of opium immediately. This was rejected, as was a second dose. *April* 2. The symptoms continue. Mr. Lawrence was sent for, and determined on operating. On dividing the sac, there was found protruding through the crural ring a portion of intestine as large as a filbert, of a reddish brown colour, and generally adhering to the sac. A director was introduced, and Gimbernat's ligament divided horizontally inwards, by means of Sir A. Cooper's curved bistoury, carried in that direction. The gut was then reduced without difficulty. The symptoms however were not relieved, and at 11 P. M. nine hours after the operation, the patient sank.

*Dissection.* On separating the edges of the wound, a knuckle of intestine, smaller and less discoloured than at the time of the operation, was found slightly adherent to the neck of the sac. It was a portion of the ileum, but little altered in structure, and on laying it open there was found no mark of stricture on its inner coat. The peritoneal coat of the smaller intestine above the hernia exhibited some marks of inflammation, but this was by no means general.

*Remarks.* This case exemplifies well the dangers of a late operation in hernia. The strangulation, or rather incarceration had existed for five days before the patient's admission into hospital, and, under such circumstances, it is not much to be wondered at that division of the stricture should give no relief. The reporter lays it down as remarkable, that, during all this time, the symptoms "had not become very urgent;" but if nausea, vomiting, hiccup, tense and painful abdomen, with a pulse 120, small and weak, and complete constipation be not urgent symptoms in hernia, we know not what are!

Two cases of strangulated hernia occurred lately at St. George's, which have some points of interest connected with them.

*Case* 1. Joseph Hynaison, æt: 36, had been subject to rupture for five or six years, but could always return it, and never wore a truss. On the 29th, whilst coughing, the gut came down with some pain, and he was unable to reduce it. The pain increased, and on the 1st of May he became affected with vomiting. He now applied to a surgeon, who bled him, administered an enema and employed the taxis, but without effect. Leeches and lotions were subsequently applied to the tumour and tartar emetic given. The vomiting persisted, and after May 2nd, he had no evacuation from the bowels, although previously he had several daily. On admission, *May 4th*, the expression was highly anxious—hands cold—pulse 108, weak and small—tongue furred and rather dry—vomiting of a green bilious matter. In the right inguinal region was a tumour of considerable size, tense, extremely painful to the touch, stretching down into the scrotum, and bearing all the marks of scrotal hernia. There was great pain and tension of the abdomen, and the patient was exceedingly restless. *Warm bath for three quarters of an hour, without the slightest relief.* At 9 P. M. the operation was resorted to as a dernier, though almost hopeless resource, by Mr. Jeffreys. It was performed in the usual way, the stricture being at the outer ring. On cutting into the sac, it was found to contain condensed omentum, of a reddish inflammatory colour, and adherent to the parietes of the sac as high as the inner ring. It was not however congested or black; in fact, there was no evidence of strangulation. The adhesions were with some difficulty broken down and the omentum returned into the abdomen. 11 P. M. Belly very painful—pulse 120 weak. *Haust. Salin.* ℥jss—*Magnes. Sulph.* ʒj. *4tis horis. May* 5, no better. *V. S. ad* ℥x. *Hirud,* xx. *abdom. Inf. Rosæ.* ℥jss, *Magn. Sulph.* ʒj. *statim.* The extremities now became quite quite cold, and at 8 P. M. he expired.

*Dissection.* The peritoneal coat of the intestines was inflamed, and the convolutions agglutinated together. A portion of omentum on the right side was injected and indurated by inflammation, whilst a little above this, and apparently in that part of the omentum which had been inclosed in the sac was an abscess containing a spoonful of good pus. Here and there the intestines were coated with layers of lymph, and in small cyst-like cavities, between these layers and the peritoneum, were small depôts of pus. In short, there was evidence of pretty acute peritoneal inflammation both on the intestines and omentum. It was evident too, that no gut had at any time been included in the stricture.

*Remarks.* This patient exhibited such decided marks of peritonitis upon his admission, that the operation was resorted to more as a forlorn hope, than as affording any great prospect of success. The "obstruction" in the bowels was here, as it often is in hernia, not the result of stricture of the canal, but of inflammation. The chances were fifty to one against recovery in this particular instance, but still in such cases, would it not be advisable to apply leeches *very freely* to the abdomen. They might keep the inflammation at bay; at any rate, they could do no harm. Let us now contrast this case with one which happened at the same hospital, and in which an early operation was performed

*Case* 2. Jonathan King, æt. 42, was admitted under Mr. Jeffreys, *April 20th,* 1827. For the last 13 months he has been subject to a rupture for which he wore a truss. Thirty-six hours ago, whilst lifting a heavy weight, the gut descended, and could not be reduced. He was taken home, a distance of some miles, in a common cart, and a surgeon called in, who attempted to bleed the patient, but without success. He then administered an enema which brought away a little feculent matter.

On admission at 12 M. there was a swelling in the right groin, about the size of a duck's egg, stretching down into the scrotum, and having all the characters of an inguinal hernia. There was considerable pain in the abdomen and tumour—not much anxiety of countenance—occasional vomiting. The taxis was tried by Mr. Jeffreys, but without effect. The man was then removed to the bath for half an hour, and ℥xxvj. of blood abstracted. Syncope occurred, he was removed to bed and the taxis employed, but again without effect. The countenance now became anxious—thirst —quick pulse. At 3 P. M. the operation was performed. On opening the sac, a considerable quantity of fluid escaped, the gut was of a dark purple colour, but by no means mortified, and it was tied down to the extremity of the sac by a band of coagulable lymph. This was torn through—the stricture, which was at the outer ring and not very tense, divided, and the gut returned without much trouble. The patient vomited and passed his fæces during the operation, but the immediate change of expression in the countenance, and relief of pain which followed its completion, were remarkable. On the third day the wound had almost entirely healed and not a bad symptom supervened.

*Remarks.* This case was well treated by Mr. Jeffreys. The changes were not rung on the taxis, and baths and bleeding, and taxis again and again, and lastly tobacco glyster—the time for operation was not parleyed away, but after a fair and ineffectual attempt at reduction, the knife was at once had recourse to. Can any thing be more illustrative of the safety and success of an early operation? We make no apology for introducing these two cases in this place, for we are sure that they are practically interesting, and bear upon an important question in surgery.

---

No. 201. July 7th. } *Compound Fracture of the Skull, without depression.*

T. P. æt. 15, was admitted into Guy's Hospital, June 22nd, under Mr. Key, with an oblique wound of the scalp on the upper and back part of the parietal bone. On introducing the probe, a fissure was detected, taking the course of the wound, about two inches in length, and quite unaccompanied with depression. The accident which occurred just previous to his admission, was occasioned by his falling with his head against the boiler of a steam engine. He was stunned by the blow. When admitted the face was pale and the pulse feeble, but no further symptoms were present. The wound was dressed with lint and adhesive plaster. In the afternoon there was some re-action with pain in the head, and pupils dilated and sluggish. A few ounces of blood were taken from the arm, and pills of colocynth and calomel exhibited. *23d.* Better—still some head-ache—pupils rather dilated. *Head to be kept cool—saline mixture, with a grain of tartar emetic, every four hours.* The wound of the scalp healed, and on July 1st, no bad symptom had appeared.

Here we see the little danger attendant on fractures of the skull without depression, provided the concussion, or its effects, be not severe. Some of that ribaldry which the reporters for the Lancet are constantly dealing forth, is tacked to this case. It may pass for wit and drollery with the writer—it is truly sickening to the reader.

*Horny Excrescence from the Neck.*—A woman, æt. 60, observed, about ten years ago, a small warty excrescence, not of a very hard texture, in the integument covering the spinous process of the second cervical vertebra. It enlarged without pain, and assumed a horny consistence. The woman was in the habit of keeping its growth under, by paring, but latterly she had allowed it to grow. She now applied to Dr. Palmer, of Arbroath, the excrescence being nine inches in length and two and half in breadth, and that gentleman, with her consent, removed it by the root. It was of the structure and form of a ram's horn, and originated from the cuticle, having little connexion with the cutis vena. No return of the disease took place. The preparation is in Panton Square.

*Lithotomy in Old Persons.* Very old men, it is well known have been cut for the stone, and with considerable success. Sir A. Cooper mentions two instances; in the one, the patient was eighty-two, and in the other, eighty-six years of age. The operation in the latter case was performed by Mr. Attenburrow, a very able surgeon of Nottingham. If, however, there be enlargement, or other disease of the prostate gland the chances in favour of the patient are much diminished. The following case which occurred at St. Thomas's Hospital, is one in point.

The patient, æt. 81, had been residing in a work-house for two years previous to his admission, and during this time his sufferings were excessive. He was anxious for an operation, and his health being tolerably good, Mr. Travers performed it on the 22nd of June. The gorget was got into the bladder, apparently without much difficulty, and a calculus about the size of a pigeon's egg extracted. Upwards of half an hour now elapsed in attempting to bring away a second calculus, which could be readily felt and grasped, but not removed on account of its magnitude. At length Mr. T. enlarging the wound in the bladder with a long curved bistoury, the stone was extracted. Considerable venous hæmorrhage followed the introduction of the gorget. On the third day, symptoms of prostration came on, and next morning the patient sank. On examination of the body a stone of some size was found in the right ureter, and the corresponding kidney wasted. The mucous membrane of the bladder was much thickened, and in points ulcerated. The prostate was enlarged and of cartilaginous hardness. In several of the veins round the neck of the bladder, calcareous matter was found.

*Inflammation of the Prepuce, with Phymosis.* W. R. æt. 24, thin and sallow, who had lived freely, and suffered from venereal complaints, observed about three weeks since, soon after suspicious connexion, a small sore on the inner surface of the frænum. To this he applied a lotion, and took calomel pills, two at night and one in the morning, which produced salivation in 48 hours. The sore, however, got worse—and four days ago, after waking, the penis became inflamed and swollen, and he could not draw back the fore-skin. *March* 16. This morning he entered Bartholomew's. A good deal of bleeding took place from the penis, giving some relief. There is phymosis, the prepuce and skin of the penis are swollen, red, and painful, and from the orifice there issues a bloody, sanious discharge. Mr. Lawrence, to liberate the inflamed parts, introduced a director, and slit up the prepuce in its whole length at the upper part. Its internal surface was highly inflamed and superficially ulcerated. The glans was inflamed also, and displayed a dirty white ragged ulceration, in which several streaks of blood appeared. The parts were excessively painful. *Calomel* gr. iv. *Jalap,* gr. xij. *immediately—bread and water poultice—salines with antimony every eight hours. To be bled if the febrile action continue.* 17*th.* He was bled to fourteen ozs. last night. The blood is inflamed. He is better in all respects. *Mag. sulph.* ʒj. *inf. ros.* ℥j. *ter quotidie—opium pill at night.* 19*th,* The sore looks sloughy. *V. S. ad* ℥xvj. *poppy poultice every four hours —salines and antimony every six. Ext. conii* gr. v. *ter quotidie.* 20*th,* Inflammation and swelling of the prepuce relieved—ulceration more healthy. Pulse full and strong, with much heat about the parts. *V. S. ad* ℥xvj. *rep. med.* From this time recovery was progressive. Both the ulceration and wound of the prepuce cicatrised, one part alone healing slowly, where the ulcerative process had extended deep.

---

No. 202, July 14th, } *Wound of the "Brachial" Vein—Gangrene of the Limb.*

W. P. æt. 35, entered St. Thomas's Hospital, June 28th, under Mr. Tyrrell, with the left arm exceedingly tense and swollen, and the skin discoloured from the shoulder downwards. It appeared that about a month previously, the man had been stabbed in the arm with a knife, that the wound did not bleed much at the time, but that in the night he lost "half a gallon" of blood of a red colour. The hæmorrhage ceased of itself, but he went to a surgeon, who applied some sticking plaster, and feeling no inconvenience he resumed his employment. On the third day, however, pain and swelling came on, and the arm had the appearance of being bruised. Twenty leeches were applied to the limb, with poppy fomentation and linseed meal poultice. Calomel and opium at bed time. Next day thirty more leeches, and a dose of laudanum at bed time. *June* 30. The arm is exceedingly swollen as high as the point of the shoulder, at which part, and as far down as the anterior fold of the axilla, the skin is of a dusky red colour: between this and the elbow it is purple. The fore-arm is less swollen, and on its outside as well as on the wrist, fore-finger, thumb and little finger, are dark vesicles. These commenced yesterday. On the outside of the biceps and a little below its middle, is a small wound, looking dark and bloody, and apparently sealed with coagulum. Pulse at the wrist not to be felt, in the other arm it is sharp and quick—tongue rather furred—countenance anxious—arm very painful in paroxysms. On the inside a diffused pulsatory motion is felt. Mr. Tyrrell imagined the case to be one of diffused aneurism from wound of the brachial artery, and considered amputation as the only effectual remedy. This, however, the state of the parts for the present forbad. In order to gain time and allow the inflammation of the skin to subside, Mr. T. directed more leeches to be applied. *July* 1*st,* Not quite so much redness about the shoulder. 2*nd,* Limb much the same. The skin having become thinner on the inside of the fore-arm, Mr. T. grew apprehensive of ulceration and hæmorrhage. Accordingly, as there was now a hand's breadth of integument below the shoulder, free from redness, he proposed amputation at the joint. In consultation, however, it was determined to remove the limb by sawing through the bone immediately beneath its tubercles.

This operation was attended with a good deal of difficulty. On making a flap of the deltoid, a great deal of extravasated blood was found, and when the artery was divided it bled so profusely notwithstanding firm pressure on the subclavian, that it was attempted to secure the axillary artery before sawing through the bone, but without effect. Even afterwards when the limb was fairly off, such was the retraction of the vessel, that upwards of half an hour elapsed before it could be tied. A temporary suture was passed through the integuments, and the patient removed to bed, but no hæmorrhage following, the wound was closed with sutures and straps made of equal parts of the adhesive and soap plasters. The constitutional irritation did not subside, but on the second morning after the operation, Mr. T. ordered the patient some porter, on account of "a want of power, indicated by a hesitation in the pulse." In the afternoon the skin was hot, and pulse rapid. Bowels freely opened with castor oil. Next evening diarrhœa came on—he had been taking eggs and porter—this last was discontinued, and six ounces of wine substituted. In a week after the operation the dressings were removed. The lower part of the wound had a sloughy appearance, but a large portion had united, and the man seemed to be doing well. About ten o'clock of the same evening a change for the worse took place—next day symptoms of sinking came on, and 6 P. M. he died. On examination of the body, no visceral disease could be detected. The soft parts about the shoulder had a dark gangrenous hue, and on cutting into them, the cellular tissue was found gorged with fluid. The limb had been inspected the day after the operation. No wound of the artery or aneurism could be discovered. The vein accompanying the artery and "*running on its outer side*," had been transfixed, and given rise to all the effusion of blood between the muscles. The gangrene was merely superficial, the muscles of the fore-arm were pale, and their connecting cellular tissue filled with fluid.

*Remarks.* This is evidently a case of gangrene, the result of erysipelatous inflammation occurring in a drunken, irritable habit. It bears no analogy whatever with those cases of true traumatic gangrene, where alone the amputation of the limb seems to promise well. In such a case as this, the *irritation* indeed is local, but the disease is constitutional, and as sure as the knife is employed, so surely, in all human probability, will inflammation and sloughing come upon the stump, if the patient survive so long. So much for the malady, but let us look a little at the *report*. The case is headed "Wound of the Brachial Vein," but in the dissection it is said that the knife "had transfixed the vein accompanying the artery, (running on its *outer* side.") Now it is scarcely necessary to tell our readers that the brachial vein lies on the *inside* of the artery, but that two small venæ comites *accompany* the artery, one on each side. The knife entered the arm on the outside of the biceps muscle, but we are not told whether it went through the muscle, or between it and the bone. To reach the brachial (or basilic) vein it had to pass the external cutaneous nerve, (which lies immediately under the biceps) the brachial artery and the median nerve, all which parts must have escaped scot-free, a circumstance not absolutely impossible certainly, but extremely improbable. But the reporter stultifies his own case, for he mentions that the vein lay on the *outside* of the artery. Now no vein whatever, as we stated before, lies on the outside of the artery, save the small vena comes which is bound up in the same sheath with it, and which it would be next to an impossibility to wound *alone*. But it appears to us, that there was an artery wounded after all. The vessel did not bleed much at first, but by secondary hæmorrhage, the man lost "half a gallon" of blood, "of a *red* colour." The wound closed, but very extensive effusion of blood took place in the limb. Are not these the common symptoms of a wounded artery? Is it likely that the puncture of one of the venæ comites should be followed by consequences like this? Is it not more likely that if not the brachial artery, at least one of its large muscular branches was wounded? Both anatomy and the symptoms *seem* to say—Yes! Be this, however, as it may, the report of the case is sadly confused and jumbled; indeed we would caution the Lancet how it meddles with aneurism; a disease which seems doomed for ever to be a tripping block to our contemporary.

*Wound of the Knee-Joint.* W. P. a cooper, æt. 19, whilst at work, about the middle of May, wounded the inside of the right knee-joint with an axe. There was a good deal of bleeding, but as far as he could tell, no discharge of synovia. Strapping and a bandage were applied, but in two or three days, he observed a considerable quantity of "joint oil" flowing from the part. He now came to Guy's Hospital as an out-patient, and was doing well, when he met with a fall, which brought on much inflammation, &c. By dint of leeches, lotions, and rest, this was greatly subdued, and on the 20th June, he entered the hospital under the care of Mr. Key. At this time the joint was much swollen, and very painful on motion, and on moving the patella, a grating sensation was distinguished. On the inside of the patella was a wound, half an inch long, filled with florid and elevated granulations, and having apparently at that time no communication with the joint. Pulse quick—tongue furred. *Hirud.* xx.—*colocynth and calomel*—*a small poultice.* Next day, lint was applied to the wound, and a bandage kept constantly wet with spirit lotion, applied to the limb, which was laid on its outer side and supported on pillows. Saline draught containing ⅙ of a grain of tartar emetic three times a day. The fluid became gradually absorbed, and on the 8th July, the joint was of its natural size, and motion performed with ease. The wound had not quite healed.

This brief case shows of what importance rest is in the management of inflamed or diseased joints. A man may leech, and lotion, and bandage as much as he will, but as long as the patient is gadding about, so long will all this treatment be labour in vain. If, however, you put that patient in bed, and keep the joint perfectly quiet by means of a roller, you will have done more towards his cure in ten days, than, under the former circumstances, you would probably have done in the same number of months. We shall on some future occasion enter into this subject more fully.

*Simple Chronic Enlargement of the Testicle.* We are confident that many testicles are extirpated in this country, and more especially on the continent, for that affection termed by Sir A. Cooper "chronic enlargement," which is entirely under the influence of mercury. The following case shows the powers of this mineral over the disease.

C. S. æt. 21, entered Guy's Hospital, under Mr. Key, on the 20th June, with the left testicle six times as large as natural. The enlargement was flattened and very hard at the sides, not so firm in front, where the skin, which was not at all discoloured, had become adherent to it. The epididymis was enlarged and hardened, as also was the cord. There was a throbbing pain in the part and in the left thigh. He stated that he had been affected with a purulent discharge from the urethra for several weeks, that, during this time, the swelling had commenced, and had gone on increasing, in spite of leeches, up to his admission. He was cupped, and took a saline draught with tartar emetic. Next day he was put upon calomel and opium. In the course of ten days, ptyalism supervened, and on the 9th July, little more than three weeks from the time of his entrance into hospital, there remained only a little hardness of the chord and epididymis.

---

No. 204, July 28th. } *Chloride of Lime in Phagedæna Gangrenosa.*

This application has been tried in a case of sloughing phagedæna at Bartholomew's, and apparently with good effect. A girl was admitted with a sore on the left labium, which included a great part of the perineum, and was covered with a dark-brown slough. The discharge was highly fetid. There was a dusky-looking circle of inflammation around—the cellular texture was puffy, and there was excessive pain. The concentrated acid was applied, and a grain of opium administered. Next day there was less general disturbance; the sore had deepened at its lower part, but the discharge was less fetid. The acid was applied a second and third time but with little good effect. The chloride of lime was now tried, and by the next day there was less irritability, and the ulcer had lost its sloughy character. Mr. Vincent not liking, it seems, to "let well alone," now applied an opium lotion, with salines internally, which had a decidedly bad effect upon the sore. The chloride of lime was resumed, and from that time the girl has been doing well.

*Prurigo cured by Colchicum.* A man, æt. 70, and upwards, was admitted with the disease in its inveterate form. Dr. Elliotson gave half a drachm of the wine of colchicum ter die. This the patient took for three weeks, at the end of which time he was dismissed cured.

*Compound Fracture of the Lower Jaw.* W. E. was admitted into St. Thomas's, on the 24th June, a carriage wheel having passed over his face. The lower jaw was broken at the left angle, and about an inch to the right of the symphysis, where the bone was so much driven in and impacted that no crepitus could be felt. Over the fracture at the angle, there was a lacerated wound, the edges of which had been brought together by sutures. The parts around were swollen—much offensive discharge—but no constitutional excitement. Upon this case Mr. Green delivered a Clinical Lecture.* He observed that pressure by tight bandages, &c. in the present instance was out of the question; for even in simple fracture, where there is no swelling, no disturbance, it is very difficult to keep the parts in apposition. The indication, then, was to give rest and allay inflammation. The patient, therefore, was fed on diluents, and not allowed to talk, whilst, at the same time, a poultice was applied to the wound, and a dose of castor oil exhibited. On the 29*th*, the discharge was healthy, and the swelling reduced, circumstances which seemed favourable for attempting union, but, as there was much thickening of the soft parts, together with swelling of the gums, the result of inflammation, Mr. G. preferred making the attempt gradually, and without violence. The teeth were brought together with silk, a compress put under the jaw, and long straps of adhesive plaster applied from under the chin to the temple, with a poultice over all. With the aid of some castor oil, all went on well, and on the 4*th*, (July) the swelling having subsided, more forcible attempts were made to bring the parts into apposition, but with only partial success. Silver wire was substituted for the silk in fastening the teeth. The patient has gone on well, and the wound is now healed.

*Affections of the Knee-Joint.* The profession may now keep jubilee, seeing what a treasure they have found in the reporter from Panton Square, where, as one of the Scotch correspondents of the Lancet justly remarks, "surgery flourisheth like a rose in the wilderness." The young gentleman takes the knee-joint under his especial protection, and some *might* say that—

> "His speech is a good sample, on the whole,
> Of rhetoric, which the learn'd call *rigmarole*."

He sets out, à l'Horace, by declaring with an air of importance, which would do no disgrace to my Lord Grizzle, that no class of diseases require "more discrimination," "greater practical knowledge," closer investigation, more *impartial* consideration, &c. than those on which he intends to enlighten us; and if this be true of joints in general, it must, of course, be so, à fortiori, of the knee. In this country, says he, they know nothing whatever of diseases of the joints; not a syllable! Xavier Bichât was the man! HE *would* have become acquainted with them, "*had* he lived," but unluckily he died, and so, of course, was unable to tell us "all about it." However, although Bichât had passed from the scene before he could investigate these affections, yet all who have written on the subject in this country have copied from him! Some people have an idea that Mr. Brodie has written not a bad book on diseases of the joints, but, save the mark! our reporter undeceives them in a twinkling. Mr. Brodie write a good book! Pooh! "his ill-gotten fame *lasted only*" till the steamers began to ply between Dover and Calais, and then, when the cockneys could take a peep at Paris, this poor gentleman's "ill-gotten fame" went out like the snuff of a candle! This is sad news, but the profession must not take it too much to heart, for, although we are little better than so many ignoramuses at present, we rejoice to add, that we shall soon be thoroughly enlightened, as our reporter means to take up the subject *himself*, and, in the present and future numbers of the Lancet, to illustrate it fully. He accordingly commences the good work by detailing, what? Why, nothing more nor less than a couple of cases of "housemaid's knee," and one of that exceedingly rare disease, synovial inflammation of the joint! Ah! Mr. Brodie, Mr. Brodie, hide thy diminished head!

* We are glad to perceive that the practice of giving Clinical Lectures is becoming so general at the Borough Hospitals. We conceive that every surgeon, who partakes of the emoluments and advantages derived from public institutions, owes it to the pupils whose money he pockets, to make them some return in kind. Every surgeon, we repeat, ought to make the more important cases which fall to his charge, the subjects of clinical instruction. If, through obstinacy or incompetency, he refuse to do this, the sooner he is out of that hospital, on which he is but an incubus, the better.

No. 205, } *Aug. 4th.* } *Ulcer of the Tongue.*

This is an interesting case. G. R. æt. 27, entered St. Thomas's, July 5th, under Mr. Travers, with an ulcer at the apex of the tongue, but more to the right than left side of it. It was larger than a shilling piece, its surface devoid of granulations, edges irregular and elevated. Four months previous to his admission he had a sore on the penis, for which he took mercury; about six weeks after this he had sore throat and eruptions, took mercury again, and got well; but, about three weeks ago, the sore on the tongue commenced. Mr. T. ordered extr. sarsapar. ʒij. Decoct. sarsapar. ℥xij Acid. nit. dilut. ʒj. Tinct. hyosciam. ʒj. A third part of the mixture thrice a day. Pil. hydr. submur. gr. v. Opii, gr. ½, at bedtime. Linimentum æruginis to the ulcer. Under this treatment, with the application, subsequently, of a solution of lunar caustic, the sore rapidly improved, and, on the 26*th*, he was made an out-patient, the sore being no more than half its original size.

*Sloughing Chancre.* M. I. æt. 22, not unhealthy-looking, a prostitute, entered Bartholomew's, under Mr. Lawrence, *April* 14*th*, with a gangrenous sore, the size of a half-crown, at the orifice of the vagina. The surface was covered with a dark, ragged slough, the discharge sanious and offensive; there was an inflammatory blush around, with acute burning pain in the parts; countenance anxious; much febrile excitement. The disease had commenced three weeks previously as a small hard pimple, which, in two days, ulcerated, when she applied to a dispensary, where she obtained some pills, one to be taken every night, with black wash for the sore. This, however, gradually spread, and became more painful, and she took six pills, with no effect. Four days before her admission, the chancre suddenly became much worse, extended rapidly, with violent pain, became foul and unhealthy, and the glands in the groin became enlarged. Mr. Lawrence hesitated, whether, in this case, he should induce a rapid ptyalism, or give anodynes; after some hesitation he decided for the latter. A lotion of equal parts of the liq. op. sedativ. and distilled water, was kept constantly applied, whilst five grains of the soap and opium pill were taken every six hours, with senna and salts to open the bowels. Next day the discharge was less offensive, the angry inflammation around diminished. *Pergat.* From this time she quickly recovered, and has left the hospital.

We think that Mr. Lawrence did very wisely in preferring the anodyne plan of treatment. The girl had been taking mercury, not very effectively, we own, but still she had been taking it, and, under its use, the chancre was spreading, the constitution beginning to suffer. Is it likely that, under these circumstances, the sore being dark and sloughy, with an angry inflammation around, and a good deal of "febrile excitement," large doses of mercury would have been of service? The anodyne treatment seems to have been clearly indicated, and its success amply justified its employment.

*Fatal Wound of the Larynx.* A middle-aged man had attempted suicide, and was brought into St. Thomas's Hospital in a state of collapse, on the evening of the 14*th July.* The wound in his throat had been stitched up by a surgeon in the neighbourhood of Peckham, and the stitches were removed by the house surgeon of the hospital, in consequence of the impeded respiration. The thyroid and cricoid cartilages were found to be wounded—the breathing was immediately relieved. On the following day, in the attempt to administer some gruel, "it was said to have escaped by the wound, which led to the supposition of there being a wound of the œsophagus." The poor man lingered a week, and then died. On dissection, the wounds of the thyroid and cricoid cartilages were found as before-mentioned; but nothing could be discovered as to the immediate cause of death. Then come the Italics and the notes of admiration of the sapient reporter. "*The œsophagus was sound throughout*! The opening through which the gruel passed *must, therefore,* have healed!" It would be exceedingly to his advantage, if the Borough-Hospital reporter trusted to his perceptions, and never gave vent to his own reflections. He may report an event that passes before his eyes correctly, as far as we know; but whenever he makes a remark, he brings forth a piece of nonsense. Did this acute anatomist not know that there was a *natural* communication between the mouth and the wound in the larynx, through which the drop of gruel might have escaped—if it did escape at all—without having recourse to the ridiculous supposition, that the œsophagus had been laid open, and healed in a few days? Did this oaf never happen to take a gulp of fluid down the *wrong way*, while at dinner, and then make it fly over his neighbours at the table? If this accident had happened to him, he would have been able to account for the phenomenon in the above case, without exposing himself to be laughed at for the absurdity of his remarks.

---

No. 206, *Aug*. 11*th*. } *Lithotomy fatal.*

J. S. a countryman, æt. 57, entered Guy's, July 14th, under Mr. Key, with symptoms of stone, of considerable standing. A sound was passed, and a calculus readily detected; his health had lately become disturbed, but his appearance, on the whole, was good. On the 24*th*, Mr. Key operated with the knife and straight staff; an artery, apparently that of the bulb, being divided, was secured by ligatures, and a stone, about the size of a halfpenny, extracted without difficulty. 26*th*. Much uneasiness about the stomach and bowels, which was attributed to flatulence, as the patient had suffered from this previously. Castor oil was given, and copious motions obtained. 27*th*. Abdomen very tympanitic—frequent eructations—pain and uneasiness of the bowels—respiration hurried—pulse moderate, but intermitting at every fifth or sixth beat. Has passed offensive fluid motions, and vomited once, the matters being mixed with much bile. *Hydr. c. cretâ, gr.* iij. *Conf. Opii, gr.* v. 4*tis horis*. 28*th*. Abdomen less distended—purging—countenance anxious—hiccup. *Leeches to the abdomen—medicines to be continued.* 30*th*. Yesterday morning, hæmorrhage, to six or eight ounces, took place from the wound, and was stopped by pressure; but, in a few hours, a second small bleeding took place. In the evening he rallied a little, but this morning the bowels are loose—the pulse feeble—skin cold. *Amm. subcarb. gr.* v. *Camph. gr.* iij. *Opii, gr.* j. *statim.* He continued, however, to sink, and died in the evening of *Aug*. 1*st*.

*Dissection*. No inflammation of the peritoneum, or cellular membrane posterior to the bladder. The viscera have an ecchymosed appearance, and the cellular tissue is lax, and readily torn. Muscular substance of the left ventricle considerably thickened, with here and there deposits of a yellow colour; loose edges of the mitral valve converted into a hard ligamentous substance. Mucous membrane of the stomach and bowels rather vascular, as was that of the bladder; cortical portion of the kidneys mottled and easily broken down.

*Remarks*. It is abundantly evident that this patient died of the shock given to the constitution by the operation, somewhat aided perhaps, by the subsequent hæmorrhage, small in quantity though it was. The symptoms throughout were not those of inflammation, but of depression, and it may admit of doubt whether support to the system might not have been substituted, with advantage, for the leeches which were employed. We have one remark to make upon the intermissions of the pulse. We are confident that this is not so untoward a symptom as some imagine, for we have again and again seen the pulse intermit after operations, and the patient do remarkably well notwithstanding. It may certainly depend at times, upon organic disease of the heart, but in very many cases we are sure it does no such thing. As the cause of death after lithotomy is a question both of great interest and importance to the surgeon, we shall here introduce a case which occurred some short time ago at St. George's Hospital.

*Case*. T. Hollamby, æt. 65, a stout hale-looking countryman, entered St. George's, under Mr. Brodie, May 9th, 1827, with all the symptoms of calculus in the bladder, on sounding which the stone was readily discovered. He had first begun to suffer from the pain in making water, &c. about a twelvemonth ago, and was affected with an old rupture on the right side, and hydrocele to boot. On the 31st, the lateral operation was performed. The perineum was exceedingly deep, the stone small and impacted behind the prostate, so that considerable difficulty was experienced in its extraction. Next day he was pretty comfortable, but on the evening of the 2nd June he became restless and anxious, and the pulse got frequent. *Haust. sennæ*, ℥ij. *statim. H. Salin. Vin. Ant. Tart.* ♏ xx—6*tis Horis*. On the 3d, the belly was rather tumid and tender on pressure—the pulse quick, and intermitting about every 8th or 10th beat. The bowels had been opened. *4th,* Much the same—manner quick and anxious—" beef-steak tongue." *3rd,* Delirious last night, and rather light-headed to-day—pain on pressing the abdomen greater—pain in the right hand and wrist, with occasionally a degree of tremor over the body; pulse quick and intermitting—tongue dry and furred—urine to-day does not flow so freely from the wound. The finger was now passed up the rectum as high as it would reach, whilst into the wound in perineo a probe-pointed bistoury, having a sliding sharp-pointed blade concealed in it, was introduced. When the probe-end had reached the cellular membrane at the neck of the bladder, the sharp point was pushed forwards, and the part laid open into the rectum, by cutting upon the finger in the gut. This was intended to give a free discharge to any matter which might have formed in the vicinity of the bladder, a circumstance not uncommon in patients with a deep perineum. The posterior part of the scrotum being swollen, an incision was made into it, and vent given to some matter in the cellular membrane. An opening which had been made yesterday, more anteriorly, was next enlarged, but here the cellular membrane contained not pus, but serum. Some vessels were divided, and easily secured. *6th,* The patient had not rallied since the operation, and at 5 o'clock this morning, he died. The urine had always flowed freely from the wound, except on the *5th,* as has been mentioned.

*Dissection.* In the cellular membrane between the prostate and rectum, and towards the left side was a pouch, the size of an egg, containing grumous and putrid blood, the cellular texture in the neighbourhood being in a sloughy state. A little below this sac was the opening into the rectum, in which there was also some putrid blood. The cut in the prostate was rather small, the gland not being by any means divided across. Nothing further of any consequence was observed about the body.

*Remarks.* There is a great similarity between this case and the preceding, the lodgment of blood in the cellular tissue around the prostate constituting the only difference. Whether the symptoms in the latter case were mainly referrible to this cause, is, we imagine, problematical, for they bore a very close resemblance indeed to those which occasionally supervene on the greater operations, and are commonly referred to the shock given to the system. The incision of the prostate was small; this we know is recommended by high authority in the profession, for by a small incision, it is said, we in a great measure avoid hæmorrhage. It may be so; but if the cut in the prostate be confined, so must be that in the cellular membrane immediately behind it, and this we should humbly conceive, would rather prevent the free escape, and, by consequence, favour the extravasation of urine or blood as the case may be.

*Aneurism of the Innominata and Subclavian.* The following case was reported in a late Number from Panton-square. Julia Barclay, married, æt. 28, came with a large pulsating tumour, situated in the course of the innominata, reaching above the sternum, extending a good deal along the clavicle, which is a good deal elevated, and involving apparently a portion of the subclavian. The pulsations were strong and distressing; circulation through the right carotid and axillary arteries unimpeded, little dyspnœa, cough, or pain; no palpitation, or other symptoms of disease of the heart, aorta, or lungs. The complaint commenced some months ago after a *bruise,* and has since increased with alarming rapidity. This was the whole of the case as reported, and it certainly looked more like a case of abscess, enlarged gland, or other tumour, than aneurism. A healthy woman of *twenty-eight,* gets a bruise on her shoulder; a swelling forms there; and proceeds rapidly, without occasioning cough, pain or dyspnœa, and without any symptoms of disease of the heart. Does this look like aneurism? We thought not; and shortly afterwards, hearing that the woman was in the Westminster Hospital, we went to see the case and satisfy our doubts as to its nature.

The woman, instead of being twenty-eight, said that she was between 32 and 33, and *looked* older than that. She had been accustomed for many years to work hard, and had that peculiar worn, and

*aneurismal* expression of countenance, which it is not, in general, difficult to recognize, though exceedingly so to describe. The complaint originated not from a bruise, but severe strain. The tumour had, as described, thrown up the clavicle and sternum too, but it was not particularly large, and on examination, the pulsatory thrill was so distinct, and so immediately beneath the fingers, as to point out at once an inordinate dilatation of the vessel, rather than an aneurismal sac built up with layers of coagulum. On applying the hand to the subclavian of the other side, the pulsation was violent and "whizzing," giving the idea of a dilatation of this vessel also. Examination by the stethoscope shewed hypertrophy of the heart, and probably dilatation of the aorta. The reporter of the case in the Lancet jumps to the conclusion, that the subclavian, or carotid, or both should be tied; after the details which have been given by us above, it is obvious that any operation of the kind would be useless or worse.

---

ANEURISM OF THE ARTERIA INNOMINATA*.

Mr. Wardrop has applied the principle of "ligature beyond the tumour" to aneurism of the innominata. This is a bold step, certainly, we only hope that it may prove as fortunate; but we shall reserve what remarks we have to make, until we have laid an abstract of the case before our readers.

Mrs. A. æt. 45, presented on the right side of the neck, an unnatural throbbing, which, on examination, was found to be owing to a pulsating tumour, the size of a turkey's egg, its base situated beneath the upper part of the sternum, and its apex rising on the inner side of the sterno-mastoid muscle. The pulsations were strong and synchronous with those of the heart—though compressible, it could not be entirely emptied, and the firmest pressure on it diminished, but did not arrest the circulation through the subclavian. This side of the neck was much less plump than the other, which in the situation of the sterno-mastoideus was more prominent than natural. No pulsation in any of the branches of the *right* carotid; very vigorous in those of the *left.* The trunk of the right carotid did pulsate very perceptibly, but this appeared to be from the impulse communicated by the tumour. Examination by percussion and the stethoscope discovered the *bruit de soufflet* for a confined space under the clavicular edge of the sterno-cleido, but no other disease in the chest. She was subject to severe pains in the *left* side of the head and neck, with a disagreeable swelling in the tumour. She had at times *great dyspnœa, increased on the slightest motion, almost to suffocation,* and obliging her to use a high pillow. Nights restless and disturbed, the patient sleeping but for a short time, and being often obliged to get out of bed. Aspect anxious, pale, and sallow; great loss of flesh; pulse frequent, full, and throbbing.

"The disease had commenced eleven months previously, with difficulty of respiration, cough, and severe pains in the chest, head, and neck." Five months afterwards, she accidentally observed, a throbbing tumour above the sternum, which in three months had decidedly increased. She was put upon digitalis, frequent small bleedings, low diet, and perfect rest, with temporary good effect, but during the last three weeks the increase of the tumour, as well as of the dyspnœa, cough, &c. had been rapid, and within a few days the apex of the swelling had become painful. Pressure to the humeral artery was applied, but the patient could not bear it for any time. Mr. Wardrop, considering, under these circumstances, that medical treatment was pretty hopeless, and taking into the account the apparent obliteration of the carotid, determined on tying the subclavian artery. Accordingly on the 6th of July this was done.

It is scarcely necessary to go over the steps of the operation, but we may mention, that great advantage was derived from placing a semi-cylindrical block of wood under the patient's neck—that two incisions were made through the integuments, one parallel to the clavicle, four inches in length, and the other parallel with and immediately above the edge of the clavicular portion of the sterno-mastoid, that instead of dissecting back successively skin and platysma myoides, and cervical fascia, these parts were cut through at once, and the incision carried

* Case where the subclavian artery was tied in an aneurism of the arteria innominata. By James Wardrop, Esq. &c. &c.—*Lancet*, No. 202.

as deep as the fibres of the sterno-cleido, thus exposing the supra-clavicular space, and dividing but once the numerous veins and arteries that lie here, the bleeding from which was inconsiderable. The operation was completed with a blunt edged silver knife, and seems to have been performed by Mr. Wardrop with his accustomed dexterity and dispatch. The pulse at the wrist ceased immediately, and there was great relief to the breathing, which for the preceding twenty-four hours had been unusually difficult. The head also was relieved, and though the size of the tumour was not perceptibly diminished, the strength of the beating was. In twenty-four hours, a slight pulsation appeared at the wrist, and has since continued. The arm did not become either numb or cold, and by the 12th July, the date of report, she had not experienced any pain in the wound, which was discharging a little pus—there had been no febrile excitement—the relief both to the breathing and pulsation had been permanent, and in short she was going on extremely well.

In No. 206 of the Lancet, the case is followed up. The patient has gone on uniformly well, the wound united almost entirely by the first intention, and on the 22nd day, the ligature came away. The tumour is much diminished in size—she has no pain in the head, cough, or dyspnœa, and she can ascend stairs with comparative ease. The patient is at present in the country for the re-establishment of her health, but proposes returning to town in the course of two months, when Mr. W. promises us the further details of the case. We forgot to mention that on the ninth day after the operation, a pulsation appeared in the right carotid artery giving rise to a variety of conjectures, which seem to us any thing but plausible.

*Remarks.* We have now to make a comment or two on this case, though it may appear somewhat daring in us, who are but little men, attempting to break a lance with a gentleman of the *calibre* of Mr. Wardrop.

We do venture to prognosticate then that this is another case of dilatation only of the artery, and further, that this dilatation is, in all probability, not confined to the innominata, but implicates the arch of the aorta, if the heart itself be free from disease. What were the first symptoms experienced by the patient? Why "difficulty of respiration —cough—and severe pains in the chest, head, and neck," all which obtained for full six months before the appearance of any local swelling or pulsation. Are these the symptoms of incipient aneurism of the innominata and of that *only?* The answer we imagine is obvious. But passing by the *history* of the complaint altogether, let us look at the patient as she presented herself to Mr. Wardrop. There was a pulsating tumour, the size of a turkey's egg in the right side of the neck, which *could be nearly emptied of its contents by pressure.* With this tumour, no larger than a turkey's egg, the patient's health was suffering greatly—the countenance was sallow, anxious—the nights sleepless —the dyspnœa such as to threaten suffocation, even on the slightest motion. Are not all this distress and dyspnœa quite out of proportion to the size of the tumour? We think there can be no question about it. We asserted that the tumour was not a *true* aneurism, and for this reason, that it was so far compressible as to admit of being nearly, though, (of course, considering the deep situation of the vessel) not entirely emptied of its contents. It is unnecessary to state that, if it were bonâ fide an aneurismal sac, built up with layers of coagulum and fibrine, this could not be the case to any such extent; besides, the history, progress, and general characters of the case bear an exact resemblance to those dilatations of the large vessels, particularly the aorta, innominata and carotids, which are so frequently met with in elderly females.

It may be said, "then the operation was not justifiable?" but softly, "my masters," we are not so sure of that. Here is an old lady, suffering excessively, and affected with a disease which must, sooner or later, run on to her destruction. She has horrible dyspnœa, which, though not entirely dependent on, is evidently kept up by, the local tumour. Under these circumstances, it becomes a question, and a very fair one, whether you shall not give her present relief by an operation, though you should ultimately fail, or even accelerate her fate. We are not as averse as some are, to desperate remedies; and if the pros and cons be *fairly* stated to a patient, and he or she wish that "something should be done,"

we know of no good reason why a surgeon should refuse to do that "something." This, however is a delicate point, and we leave it to abler heads and *hands* to decide.

This paper was just in the hands of the printer, when we noticed a direct confirmation of the opinions it expressed, in a letter from Dr. Barry to Mr. Wardrop, published in No. 208 of the Lancet. Dr. B. was requested by Mr. W. to see the patient, which he did, at her own house, on the forenoon of the 6th ult. in the presence of her husband. The results of his examination by the stethoscope and otherwise, are communicated in the above-mentioned letter. We cannot insert more than the Doctor's *diagnosis* and *prognosis*, which we shall give in his own words.

"*Diagnosis*.—From the impulse felt under and below the right clavicle, and from its absence in the præcordial regions, the impelled body must be situated above the base of the heart. From the situation of the tumour, from the results afforded by percussion, the arteria innominata must be considerably dilated. From the wheezing, (rale sibilante,) from the impulse synchronous with the pulse, from the '*bruit de scie*,' the aorta within the pericardium, and at its arch, is most probably dilated, and presses upon some of the bronchi. The regularity of the motions of the heart, and the total absence of palpitations, would seem to show that the valves are perfect. From the increased size of the left carotid, and from the absence of pulsation in the right, I would say that the former is uniformly dilated and the latter obliterated, or nearly so.

"*Prognosis*.—From all the above considerations, and from others not strictly mechanical, I would say, that this case must terminate fatally, but perhaps at a very distant period, and that no operation on the *right carotid* can be of service." 656.

Here, then, we see that Dr. Barry has confirmed, by the stethoscope, what we had only surmised from the history and symptoms; to wit, that the innominata is dilated, and that the aorta, within the pericardium, and at its arch is probably dilated also. Want of space compels us to defer the consideration of the operation itself, ligature of the subclavian, to some future opportunity.

*Wound of the Throat—Death by Syncope*. A case is reported in No. 208 of the Lancet, from Bartholomew's Hospital, which is calculated to excite some unpleasant sensations. A man had attempted suicide, but the wound of the throat glanced in between the epiglottis and rima glottidis, so that, in fact, the larynx was not opened at all, though it is called by the reporter "*a large transverse opening in the larynx! !*" But let that pass. The man had wounded himself in the arm also, and, on this wound, erysipelas supervened. In the course of a fortnight, Mr. Lawrence deemed it necessary to make two incisions in the fore-arm, "*extending nearly the length of the limb.*" Blood, to the amount of 20 ounces, flowed from these incisions, when syncope took place, from which the patient never recovered! Mr. Lawrence observed to the pupils, that "the fatal event of this case was one of those unusual occurrences, which neither the circumstances of the patient before, nor the examination after death, could elucidate." This may be true—and Mr. L. may be right in saying that he would "adopt exactly the same practice in another similar case." But would Mr. Lawrence allow blood to flow from incisions made in an erysipelatous limb till syncope took place? We rather think he would not, from an expression which incidentally appears on the record. "But he conceived that the present instance would suggest the *salutary caution* of attending closely to the state of the circulation on these occasions." Now this "*salutary caution*" either *was*, or was *not* taken. If it was taken, the event shews that it was not efficacious, and, consequently, that the practice was dangerous. If it was *not* taken, the fair inference is, that the patient's death was occasioned by the syncope, which should have been prevented if possible. Utrum horum mavis accipe.

We would just suggest, that the circumstance of erysipelas being present in a patient, ought to prove a check to our proceeding to such lengths in depletion, whether from a vein or incisions, as to induce syncope. The latter taking place in the *horizontal* position, is a formidable phenomenon at all times—and especially in a patient labouring under erysipelas, and all that mental depression and nervous irritation which generally attend the act of suicide.

